# Anticholinergic Toxicity in the Emergency Department

**DOI:** 10.21980/J8D07Z

**Published:** 2023-01-31

**Authors:** C Eric McCoy, Reid Honda

**Affiliations:** * University of California, Irvine, Department of Emergency Medicine, Orange, CA; ^ Queens Medical Center, Department of Emergency Medicine, Honolulu, HI

## Abstract

**Audience:**

Emergency medicine residents, internal medicine residents, family medicine residents, community physicians, pediatricians, toxicology fellows

**Introduction:**

There are over 600 compounds which contain anticholinergic properties.1 Medications with anticholinergic properties include antihistamines, atropine, tricyclic antidepressants, antipsychotics, topical mydriatics, antispasmodics, sleep aids, and cold preparations. 1–4 Plants that possess anticholinergic properties such as jimson weed, and street drugs cut with anticholinergics such as scopolamine are sources of accidental or intentional ingestion.1,2,4 Anticholinergic toxicity can cause a myriad of signs and symptoms, including agitation, seizures, hyperthermia, cardiac dysrhythmias, and death. Since poisoning from anticholinergic medications is frequently encountered in the emergency department, is it essential that emergency physicians be familiar with how to manage this toxidrome. This simulation case will allow the learner to evaluate and manage a patient presenting with anticholinergic toxicity.

**Educational Objectives:**

By the end of this simulation case, learners will be able to: 1) describe the classic clinical presentation of anticholinergic toxicity, 2) discuss common medications and substances that may lead to anticholinergic toxicity, 3) recognize the electrocardiogram (ECG) findings in anticholinergic toxicity that require specific therapy, and 4) review the management of anticholinergic toxicity.

**Educational Methods:**

This simulation is taught using a high- or moderate-fidelity manikin.

**Research Methods:**

The educational content was evaluated by the learners immediately after completion and debriefing of the scenario. This case was initially piloted with approximately twenty emergency medicine residents. The group was comprised of first, second-, and third-year residents from a three-year emergency medicine residency. The efficacy of the content was assessed by oral feedback.

**Results:**

Overall, the case was well received by learners, who felt it was useful and were engaged throughout the session. The overall feedback was positive and the case was well-received by learners.

**Discussion:**

This scenario was eventually tested on over 100 learners over the course of several years, and the overall feedback was positive. It was found to be effective when debriefing sessions using various debriefing techniques (such as advocacy/inquiry) were utilized to discuss both the learners’ performance in the case, as well as the debriefing pearls (located at the end of this manuscript).

**Topics:**

Anticholinergic toxicity, altered mental status, toxicology.

## USER GUIDE

**Table t1-jetem-8-1-s25:** 

List of Resources:	
Abstract	25
User Guide	27
Instructor Materials	28
Operator Materials	37
Debriefing and Evaluation Pearls	40
Simulation Assessment	43

[Table t1-jetem-8-1-s25] **Learner Audience:**

Interns, junior residents, senior residents, community physicians, pediatricians, toxicology fellows

**Time Required for Implementation:**

Instructor Preparation: 20–30 minutes

Time for case: 10–15 minutes

Time for debriefing: 15–30 minutes

**Recommended Number of Learners per Instructor:** 3–5

**Topics:**

Anticholinergic toxicity, altered mental status, toxicology.

**Objectives:**

By the end of this simulation case, learners will be able to:

1. Describe the classic clinical presentation of anticholinergic toxicity
2. Discuss common medications and substances that may lead to anticholinergic toxicity
3. Recognize the ECG findings in anticholinergic toxicity that require specific therapy
4. Review the management of anticholinergic toxicity

### Linked objectives and methods

During this simulation, the participants will need to make the diagnosis of anticholinergic toxicity in a critically ill patient presenting with undifferentiated altered mental status (objective 1). If a thorough history and collateral information is obtained, the participants will realize that the patient overdosed on diphenhydramine and jimson weed (objective 2). During the case, the patient will be tachycardic with a widened QRS interval, requiring treatment with sodium bicarbonate (objective 3). In addition, the patient will require treatment with benzodiazepines, supportive care, and potential toxicology/poison center consultation (objective 4).

### Recommended pre-reading for instructor

- Fulton J, Nelson L. Anticholinergics. In: Adams J, ed. *Emergency Medicine: Clinical Essentials*. 2^nd^ Edition. Philadelphia, PA: Elsevier; 2012:1240–1245.
- Wax PM. Anticholinergic toxicity. In: Tintinalli JE, Stapczynski J, Ma O, Cline DM,, Meckler GD, eds. *Tintinalli’s Emergency Medicine: A Comprehensive Study Guide. 8^th^ ed*. New York, NY: McGraw-Hill; 2016:1143–1146.

### Results and tips for successful implementation

This simulation scenario is best implemented in an educational environment that includes high- or moderate-fidelity simulation resources along with monitors that will allow the learner(s) to independently interpret vital signs and visual stimuli (ie, a simulation center or equivalent educational space). This scenario was tested on more than 100 learners over the course of several years. Performance is best measured with a critical actions checklist that can be used during the real-time assessment of the simulation scenario by proctors with the primary purpose of participant evaluation.

The educational content was evaluated by the learners immediately after completion and debriefing of the scenario. This case was initially piloted with approximately twenty emergency medicine residents. The group was comprised of first, second-, and third-year residents from a three-year emergency medicine residency. The efficacy of the content was assessed by oral feedback. Overall, the case was well received by learners, who felt it was useful and were engaged throughout the session. The overall feedback was positive, and the case was well-received by learners.

## Supplementary Information















## Figures and Tables

**Table t2-jetem-8-1-s25:** 

#1	Electrocardiogram (ECG) showing sinus tachycardia with widened QRS interval (>100ms)
#2	Chest Radiograph (CXR)
#3	Complete blood count (CBC)
#4	Comprehensive metabolic panel (CMP)
#5	Urinalysis (UA) and Urine Drug Screen

**Table t3-jetem-8-1-s25:** 

**Vitals:**	Heart Rate (HR) 137	Blood Pressure (BP) 167/95	Respiratory Rate (RR) 22
	Temperature (T) 38.7°C	Oxygen Saturation (O_2_Sat) 98% on room air (RA)	

**Figure f1-jetem-8-1-s25:**
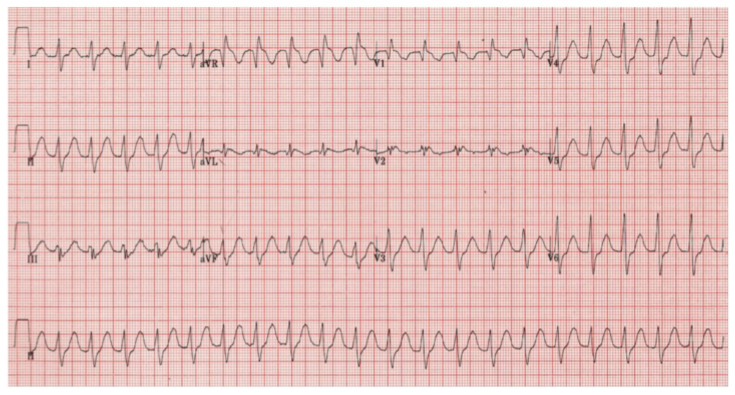
*Electrocardiogram (ECG) –* sinus tachycardia with widened QRS interval Image source: (author’s own image)

**Figure f2-jetem-8-1-s25:**
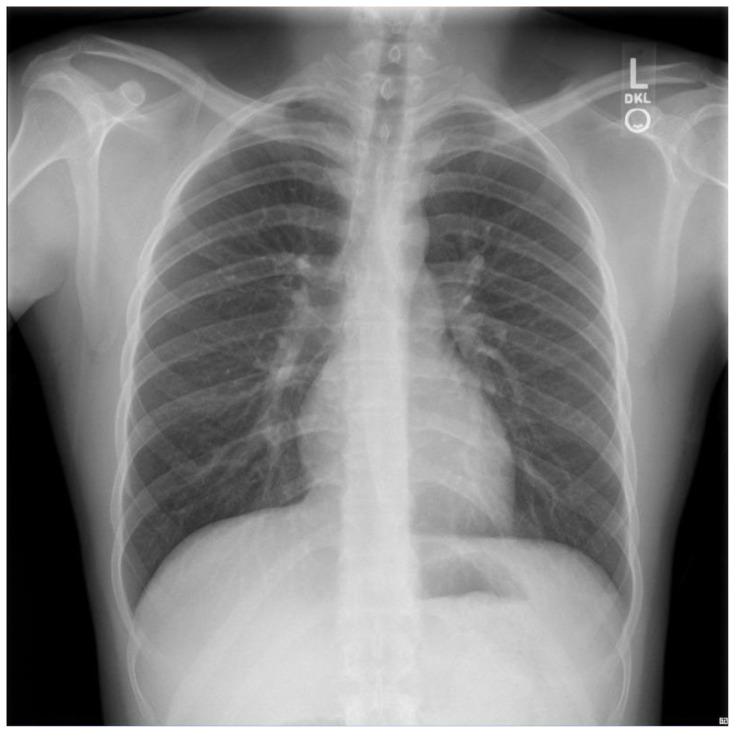
Chest Radiograph (CXR) Image source: Author’s own image

**Table t4-jetem-8-1-s25:** 

*Complete blood count (CBC)*	
White blood count (WBC)	15.0 ×1000/mm^3^
Hemoglobin (Hgb)	14.0 g/dL
Hematocrit (HCT)	40.0%
Platelet (Plt)	250 ×1000/mm^3^

**Table t5-jetem-8-1-s25:** 

*Comprehensive metabolic panel (BMP)*	
Sodium	140 mEq/L
Chloride	100 mEq/L
Potassium	4.7 mEq/L
Bicarbonate (HCO_3_)	19 mEq/L
Anion Gab	21 mEq/L
Blood Urea Nitrogen (BUN)	30 mg/dL
Creatine (Cr)	1.1 mg/dL
Glucose	100 mg/dL
Calcium	9.5 mg/dL
Total Protein	7.0 g/dL
Albumin	40 units/L
Alkaline Phoshatase	100 units/L
Aspartate aminotransferase (AST)	90 units/L
Alanine aminotransferase (ALT)	60 units/L

**Table t6-jetem-8-1-s25:** 

*Urinalysis (UA)*	
Color	yellow
Appearance	clear
Specific gravity	1.010
pH	7.0
Glucose	negative
Ketones	negative
Hemoglobin	negative
Leukocyte esterase	negative
Nitrites	negative
White blood cells (WBC)	0 WBCs/high powered field (HPF)
Red blood cells (RBC)	0 RBCs/HPF
Squamous epithelial cells	1 cells/HPF
Bacteria	negative

**Table t7-jetem-8-1-s25:** 

*Urine Toxicology Screen:*	
Tetrahydrocannabinol (THC)	None Detected
Phencyclidine (PCP)	None Detected
Benzodiazepines	None Detected
Barbiturates	None Detected
Methadone	None Detected
Cocaine	None Detected
Amphetamines	None Detected
MDMA	None Detected

**Table t8-jetem-8-1-s25:** 

Minute (state)	Participant action/ trigger	Patient status (simulator response) & operator prompts	Monitor display (vital signs)
0:00 (Baseline)	Assess ABCs Attach patient to cardiac monitor and pulse ox Obtain initial set of vitals	Patient arrives confused and intermittently agitated.	T 38.7°C HR 137 BP 167/95 RR 22 O2 98% on RA
02:00	Obtain history from paramedics Obtain history from patient’s friend Perform physical exam Obtain finger-stick glucose	If asked, paramedics will state that patient was found wandering around mobile home park confused. If asked, patient’s friend will state that patient was making tea with jimson weed and liquid diphenhydramine, which he consumed prior to becoming altered. Physical exam will be significant for dry, warm, flushed skin, mydriasis, tachycardia, and confusion. Finger-stick glucose will be normal.	T 38.7°C HR 137 BP 167/95 RR 22 O2 98% on RA
04:00	Make diagnosis of anticholinergic toxicity Obtain IV access Order labs Order ECG	Patient’s agitation may be treated with benzodiazepines. If benzodiazepine administered: Patient’s agitation and tachycardia will improve. If diagnosis of anticholinergic toxicity not made by this time and patient not given benzodiazepine, patient will become increasingly agitated and have a seizure. If patient has seizure, it will resolve with benzodiazepine administration. If benzodiazepine is given, vitals will stabilize, and move on to time 6:00. If benzodiazepine is not given, patient will go into cardiac arrest and expire.	If benzo given: HR 115 BP 140/70 RR 18 O2 98% on RA If benzo not given, seizure: HR 150 BP 170/90 RR 28 O2 88% on RA If benzo given, seizure resolves: HR 120 BP 140/90 RR 18 O2 98% on RA
06:00	Poison control/ toxicology consult should be obtained Lab results will return	ECG is available demonstrating sinus tachycardia with a QRS >100msec. Participants should recognize wide QRS complex and initiate treatment with sodium bicarbonate. If poison control/toxicology consult obtained, they will prompt participants to review ECG and give sodium bicarbonate. They will also recommend that activated charcoal be given. They will recommend against physostigmine, because patient’s QRS is >100ms. **If sodium bicarb is given:** Patient’s vital signs will remain stable, proceed to time 10:00 **If sodium bicarb not given:** Patient will go into Ventricular fibrillation. If ACLS started, patient will not obtain return of spontaneous circulation (ROSC) until sodium bicarb is given. If sodium bicarb given, patient will obtain ROSC, proceed to time 10:00. If sodium bicarb still not administered, patient will expire and case will end. If physostigmine is given, patient will decompensate into cardiac arrest and case will end.	If sodium bicarb given: HR 110 BP 130/60 RR 18 O2 98% on RA If sodium bicarb not given: HR v Fib BP 0 RR 0 O2 undetectable
10:00	Admit the patient to the ICU	If fluids, benzodiazepines, and sodium bicarb given, patient’s agitation and vital signs will improve, and patient should be admitted to the ICU. If no treatment was started, and/or if the diagnosis of anticholinergic toxicity was not made, the patient will have further seizures requiring intubation, and will ultimately go into cardiac arrest and expire.	HR 90 BP 140/160 RR 18 O2 98% on RA

**Table t9-jetem-8-1-s25:** 

**“Mad as a hatter”**	Blockage of CNS muscarinic receptors	Delirium, hallucinations, anxiety, agitation, seizure CNS symptoms most worrisome, considered “severe” toxicity
**“Blind as a bat”**	Pupillary dilation and ineffective accommodation	Blurry vision
**“Dry as a bone”**	Blockage of sweat gland muscarinic receptors → lack of sweating	Dry skin
**“Hot as a hare”**	Lack of sweating → anhidrotic hyperthermia	Hyperthermia
**“Red as a beet”**	Loss of sweat production → Body attempts to compensate by cutaneous vasodilation to dissipate heat	Red, flushed skin
**“Full as a flask”**	Detrusor muscle of bladder and urethral sphincter under muscarinic control	Urinary retention
**Tachycardia**	Caused by inhibition of muscarinic receptors on vagus nerve^4^ Certain agents may also produce sodium channel blockade (eg, TCAs--tricyclic antidepressants, diphenhydramine)^3^,^4^	Tachycardia Dysrhythmias (widened QRS, prolonged QT)

**Table t10-jetem-8-1-s25:** 

**Critical Actions:** Assess airway, breathing, and circulation (ABCs) Place patient on cardiac monitors and pulse oximeter, and obtain initial set of vital signs Establish two large bore IV lines Administer IV fluids (at least 1 liter) Make the diagnosis of anticholinergic toxicity Contact the poison control center/obtain toxicologist consult Give sodium bicarbonate for QRS >100msec on ECG Give benzodiazepines to control agitation Admit patient to the intensive care unit	0:00

**Table t11-jetem-8-1-s25:** 

	Milestone	Did not achieve level 1	Level 1	Level 2	Level 3
1	**Emergency Stabilization (PC1)**	Did not achieve Level 1	Recognizes abnormal vital signs	Recognizes an unstable patient, requiring intervention Performs primary assessment Discerns data to formulate a diagnostic impression/plan	Manages and prioritizes critical actions in a critically ill patient Reassesses after implementing a stabilizing intervention
2	**Performance of focused history and physical (PC2)**	Did not achieve Level 1	Performs a reliable, comprehensive history and physical exam	Performs and communicates a focused history and physical exam based on chief complaint and urgent issues	Prioritizes essential components of history and physical exam given dynamic circumstances
3	**Diagnostic studies (PC3)**	Did not achieve Level 1	Determines the necessity of diagnostic studies	Orders appropriate diagnostic studies. Performs appropriate bedside diagnostic studies/procedures	Prioritizes essential testing Interprets results of diagnostic studies Reviews risks, benefits, contraindications, and alternatives to a diagnostic study or procedure
4	**Diagnosis (PC4)**	Did not achieve Level 1	Considers a list of potential diagnoses	Considers an appropriate list of potential diagnosis May or may not make correct diagnosis	Makes the appropriate diagnosis Considers other potential diagnoses, avoiding premature closure
5	**Pharmacotherapy (PC5)**	Did not achieve Level 1	Asks patient for drug allergies	Selects an medication for therapeutic intervention, consider potential adverse effects	Selects the most appropriate medication and understands mechanism of action, effect, and potential side effects Considers and recognizes drug-drug interactions
6	**Observation and reassessment (PC6)**	Did not achieve Level 1 Reevaluates patient at least one time during case	Reevaluates patient after most therapeutic interventions	Consistently evaluates the effectiveness of therapies at appropriate intervals	
7	**Disposition (PC7)**	Did not achieve Level 1	Appropriately selects whether to admit or discharge the patient	Appropriately selects whether to admit or discharge Involves the expertise of some of the appropriate specialists	Educates the patient appropriately about their disposition Assigns patient to an appropriate level of care (ICU/Tele/Floor) Involves expertise of all appropriate specialists
9	**General Approach to Procedures (PC9)**	Did not achieve Level 1	Identifies pertinent anatomy and physiology for a procedure Uses appropriate Universal Precautions	Obtains informed consent Knows indications, contraindications, anatomic landmarks, equipment, anesthetic and procedural technique, and potential complications for common ED procedures	Determines a back-up strategy if initial attempts are unsuccessful Correctly interprets results of diagnostic procedure
20	**Professional Values (PROF1)**	Did not achieve Level 1	Demonstrates caring, honest behavior	Exhibits compassion, respect, sensitivity and responsiveness	Develops alternative care plans when patients’ personal beliefs and decisions preclude standard care
22	**Patient centered communication (ICS1)**	Did not achieve level 1	Establishes rapport and demonstrates empathy to patient (and family) Listens effectively	Elicits patient’s reason for seeking health care	Manages patient expectations in a manner that minimizes potential for stress, conflict, and misunderstanding. Effectively communicates with vulnerable populations, (at risk patients and families)
23	**Team management (ICS2)**	Did not achieve level 1	Recognizes other members of the patient care team during case (nurse, techs)	Communicates pertinent information to other healthcare colleagues	Communicates a clear, succinct, and appropriate handoff with specialists and other colleagues Communicates effectively with ancillary staff

## INSTRUCTOR MATERIALS

**Case Title:** Anticholinergic Toxicity in the Emergency Department

**Case Description & Diagnosis (short synopsis):** An 18-year-old male is brought in by ambulance after being found altered and wandering around a mobile home park. Upon arrival to the emergency department, he is confused, combative, febrile, and tachycardic. Upon further questioning of his friend at bedside, it is discovered that the patient and his friends were drinking tea made out of jimson weed and liquid diphenhydramine, as part of a viral online social media challenge, prior to his altered level of consciousness. The patient is ultimately found to be presenting with an anticholinergic toxidrome secondary to consumption of jimson weed and diphenhydramine.

**Equipment or Props Needed:**

- High- (or moderate-) fidelity simulation mannequin
- Cardiac monitor, pulse oximeter
- Blood pressure cuff
- Intravenous (IV) catheter and lines
- Crash cart with defibrillator
- Bag valve mask
- Intubation equipment (endotracheal tube, laryngoscope)
- Syringes to simulate medications
- Normal saline

**Confederates needed:**

- Nurse to assist with management of patient
- Paramedics who bring patient into emergency department (this can be played by simulation operator overhead)
- Friend who arrives with patient and paramedics (this can be played by simulation operator overhead)

**Stimulus Inventory:** [Table t2-jetem-8-1-s25]

**Background and brief information:** The scenario takes place in an emergency department at a community hospital. The patient is an 18-year-old male brought in by paramedics with a chief complaint of altered mental status.

**Initial presentation:** The patient is confused and intermittently agitated upon arrival. His vital signs are significant for a fever and tachycardia. His physical exam is significant for dry and flushed skin, dilated pupils, and altered mental status. He is unable to provide further history.

**How the scene unfolds:** The patient arrives to the emergency department confused and unable to provide further history. If asked, the paramedics will report that the patient was found wandering about his mobile home park confused. They will report that the patient was tachycardic on scene, and that his finger-stick blood glucose was normal. They will state that the patient was intermittently combative en route, and would not cooperate with the non-rebreather face mask that they attempted to place on him. If the participants ask the patient’s friend at bedside for more information, he will state that the patient and some friends were taking part in a viral online social media challenge, and were drinking a tea made out of jimson weed and liquid diphenhydramine, which the patient consumed shortly before becoming altered.

On arrival, the patient is tachycardic and hyperthermic. His physical exam is significant for dry, flushed skin, dilated pupils, and altered mental status. The patient’s ECG will be significant for tachycardia with a QRS >100ms. The participants should make the diagnosis of anticholinergic toxicity. The participants may initiate treatment with activated charcoal. The participants should also contact the poison control center. They may control the patient’s agitation using benzodiazepines. If the diagnosis of anticholinergic toxidrome is delayed and benzodiazepines are not administered, the patient will become increasingly agitated and will have a seizure.

The participants should recognize the concerning finding of the QRS >100msec on ECG, and should administer sodium bicarbonate. If the participants do not recognize this, but consult the poison control center, the toxicologist will prompt them to review the ECG and initiate sodium bicarbonate. If sodium bicarbonate is not given, the patient will go into ventricular fibrillation. If the participants administer physostigmine, the patient will go into cardiac arrest. The patient should ultimately be admitted to the intensive care unit for further observation and management.

**Critical actions:**

1. Assess airway, breathing, and circulation (ABCs)
2. Place patient on cardiac monitors and pulse oximeter, and obtain initial set of vital signs
3. Establish two large bore IV lines
4. Administer IV fluids (at least 1 liter)
5. Make the diagnosis of anticholinergic toxicity
6. Contact the poison control center/obtain toxicologist consult
7. Give sodium bicarbonate for QRS >100msec on ECG
8. Give benzodiazepines to control agitation
9. Admit patient to the intensive care unit

**Case Title:** Anticholinergic Toxicity in the Emergency Department

**Chief Complaint:** The patient is an 18-year-old male presenting with a chief complaint of altered mental status[Table t3-jetem-8-1-s25]

**General Appearance:** Appears stated age, in moderate distress

**Primary Survey:**

- **Airway:** Patent
- **Breathing:** Clear to auscultation bilaterally, slightly tachypneic
- **Circulation:** 2+ femoral pulses bilaterally

**History:**

- **History of present illness:** The patient is an 18-year-old male who presents brought in by paramedics with altered mental status. The patient will be too altered to provide any history. If asked, the paramedics will report that the patient was found wandering about a mobile home park altered. They will report that he was tachycardic in the field, and that his finger-stick blood glucose was normal. They will state that he was intermittently agitated en route. If the friend at bedside is questioned, he will state that the patient and his friends were drinking tea made out of jimson weed and liquid diphenhydramine earlier in the day.
- **Past medical history:** Unable to obtain from patient due to altered mental status
- **Past surgical history:** Unable to obtain from patient due to altered mental status
- **Patient’s medications:** Unable to obtain from patient due to altered mental status
- **Allergies:** Unable to obtain from patient due to altered mental status
- **Social history:** Unable to obtain from patient due to altered mental status
- **Family history:** Unable to obtain from patient due to altered mental status

**Secondary Survey/Physical Examination:**

- **General appearance:** Awake, confused, dazed look on face, appears to be responding to internal stimuli
- HEENT):
  ○ **Head:** No external signs of trauma
  ○ **Eyes:** Pupils dilated 8mm bilaterally, extra-ocular movements intact
  ○ **Ears:** within normal limits (WNL)
  ○ **Nose:** WNL
  ○ **Throat:** No tonsillar exudates, no erythema
- **Neck:** No midline C-spine tenderness, full range of motion, no swelling, trachea midline
- **Heart:** Tachycardic, no audible murmurs
- **Lungs:** Clear to auscultation bilaterally, slightly tachypneic
- **Abdominal/gastrointestinal:** Soft, non-distended, non-tender to palpation, no guarding, no rigidity. Slight fullness noted to suprapubic region. Decreased bowel sounds.
- **Genitourinary:** Normal appearing external male genitalia
- **Rectal:** Normal tone, no melena, no gross blood
- **Extremities:** No gross deformities. No lower extremity edema.
- **Back:** No thoracic or lumbar spine tenderness, no step-offs
- **Neuro:** Awake. Not answering questions appropriately. No facial droop. Intermittently following commands. Intermittently agitated. Appears to be responding to internal stimuli, reaching for things in the air. Moves all four extremities, no significant weakness noted on allowable exam.
- **Skin:** Dry, flushed skin, warm to touch, no diaphoresis
- Lymph: WNL
- **Psych:** Intermittently agitated, appears to be responding to internal stimuli

**Results:** [Fig f1-jetem-8-1-s25] [Fig f2-jetem-8-1-s25] [Table t4-jetem-8-1-s25] [Table t5-jetem-8-1-s25] [Table t6-jetem-8-1-s25] [Table t7-jetem-8-1-s25]

## OPERATOR MATERIALS

**SIMULATION EVENTS TABLE:[Table t8-jetem-8-1-s25]**

**Diagnosis:**

Anticholinergic toxicity secondary to jimson weed ingestion

**Disposition:**

Intensive care unit

## DEBRIEFING AND EVALUATION PEARLS

### Anticholinergic Toxicity

- Introduction:
  ○ There are over 600 compounds which contain anticholinergic properties.^1^ Medications with anticholinergic properties include antihistamines, atropine, tricyclic antidepressants, antipsychotics, topical mydriatics, antispasmodics, sleep aids, and cold preparations.^1^,^2^,^3^,^4^ Plants such as jimson weed (*datura stramonium*) and deadly nightshade (*atropa belladonna*) also have anticholinergic properties and are a common source of accidental or intentional ingestion.^1^,^2^,^4^ Street drugs such as heroin and cocaine have been known to be cut with anticholinergics such as scopolamine. 1,2 Thus, it is not surprising that anticholinergic poisonings are frequently seen in the emergency department, and recognition of the anticholinergic toxidrome is a necessary clinical skill.^1^,^4^
- Pathophysiology and Clinical Manifestation:
  ○ Muscarinic acetylcholine receptors are located in the central nervous system, heart, smooth muscle, secretory glands, and ciliary body of the eye.^1^
  ○ Normally, the neurotransmitter acetylcholine binds to these muscarinic receptors^1^
  ○ Anticholinergic drugs competitively inhibit this binding, producing the following clinical effects:[Table t9-jetem-8-1-s25]

- **Management:**
  ○ Stabilization of airway, breathing, and circulation is paramount, as well as initiating IV access and placing patient on cardiac monitoring and pulse oximetry. 1
  ○ **Decontamination:**
    ▪ Activated charcoal may be considered as long as patient is able to protect their airway or if they are intubated, though patients shouldn’t be intubated solely for the purpose of administering activated charcoal. 1,2,4
    ▪ May be considered even if ingestion occurred more than one hour prior, because anticholinergics decrease gastrointestinal motility. 2,4
  ○ **Benzodiazepines:**
    ▪ Should be used intravenously to treat agitation and seizures. 1,2,4
  ○ **Sodium bicarbonate:**
    ▪ Administered to treat wide complex dysrhythmias secondary to sodium channel blockade. 1,2
    ▪ Indicated if QRS is greater than 100–120 milliseconds. 4
  ○ **Physostigmine:**
    ▪ Physostigmine is an acetylcholinesterase inhibitor → increases amount of acetylcholine accumulating in synapse → overcomes effects of anticholinergic agents. 1,4
    ▪ May be indicated when patients have significant central anticholinergic toxicity. 1
      - Beneficial in treatment of agitation or delirium. 4
    ▪ Use is controversial:
      - May cause cholinergic toxicity if patient is not poisoned with an anticholinergic substance, or if dosing is incorrect. 1
      - Case reports describing patients who had a wide QRS interval after TCA-poisoning who developed asystole after being treated with physostigmine. 1
    ▪ Should not be given if a condition other than pure anticholinergic poisoning is suspected (eg, tricyclic antidepressant overdose), or if QRS interval is at or above 100 msec. 1,4
    ▪ If considering using physostigmine, consult a toxicologist.
- **Disposition:**
  ○ Asymptomatic patients → Should receive activated charcoal, and be observed for at least six hours in the emergency department and may be discharged if they continue to be asymptomatic. 1
  ○ Mild anticholinergic toxicity → Should receive activated charcoal, be treated with benzodiazepines if required, and observed for six hours for resolution of symptoms. Discharge can be considered if symptoms resolve; otherwise, they should be admitted for observation. 1
  ○ Severe anticholinergic toxicity, and those treated with physostigmine → Should be admitted to an intensive care unit.^1^

## SIMULATION ASSESSMENT

*Anticholinergic Toxicity in the Emergency Department*

Learner: _________________________________________

***Assessment Timeline***

This timeline is to help observers assess their learners. It allows observer to make notes on when learners performed various tasks, which can help guide debriefing discussion.[Table t10-jetem-8-1-s25]

**Critical Actions:**

□ Assess airway, breathing, and circulation (ABCs)
□ Place patient on cardiac monitors and pulse oximeter, and obtain initial set of vital signs
□ Establish two large bore IV lines
□ Administer IV fluids (at least 1 liter)
□ Make the diagnosis of anticholinergic toxicity
□ Contact the poison control center/obtain toxicologist consult
□ Give sodium bicarbonate for QRS >100msec on ECG
□ Give benzodiazepines to control agitation
□ Admit patient to the intensive care unit

**Summative and formative comments:**

**Milestones assessment:** [Table t11-jetem-8-1-s25]
