# Supplementary material for: Anticholinergic Toxicity in the Emergency Department
Source: J Educ Teach Emerg Med. 2023 Jan 31;8(1):S25–47. doi: 10.21980/J8D07Z (PMC10332772; doi:10.21980/J8D07Z)
Supplement: Supplementary file 1 [file jetem-8-1-S25-supp1.pptx]

## Slide 1
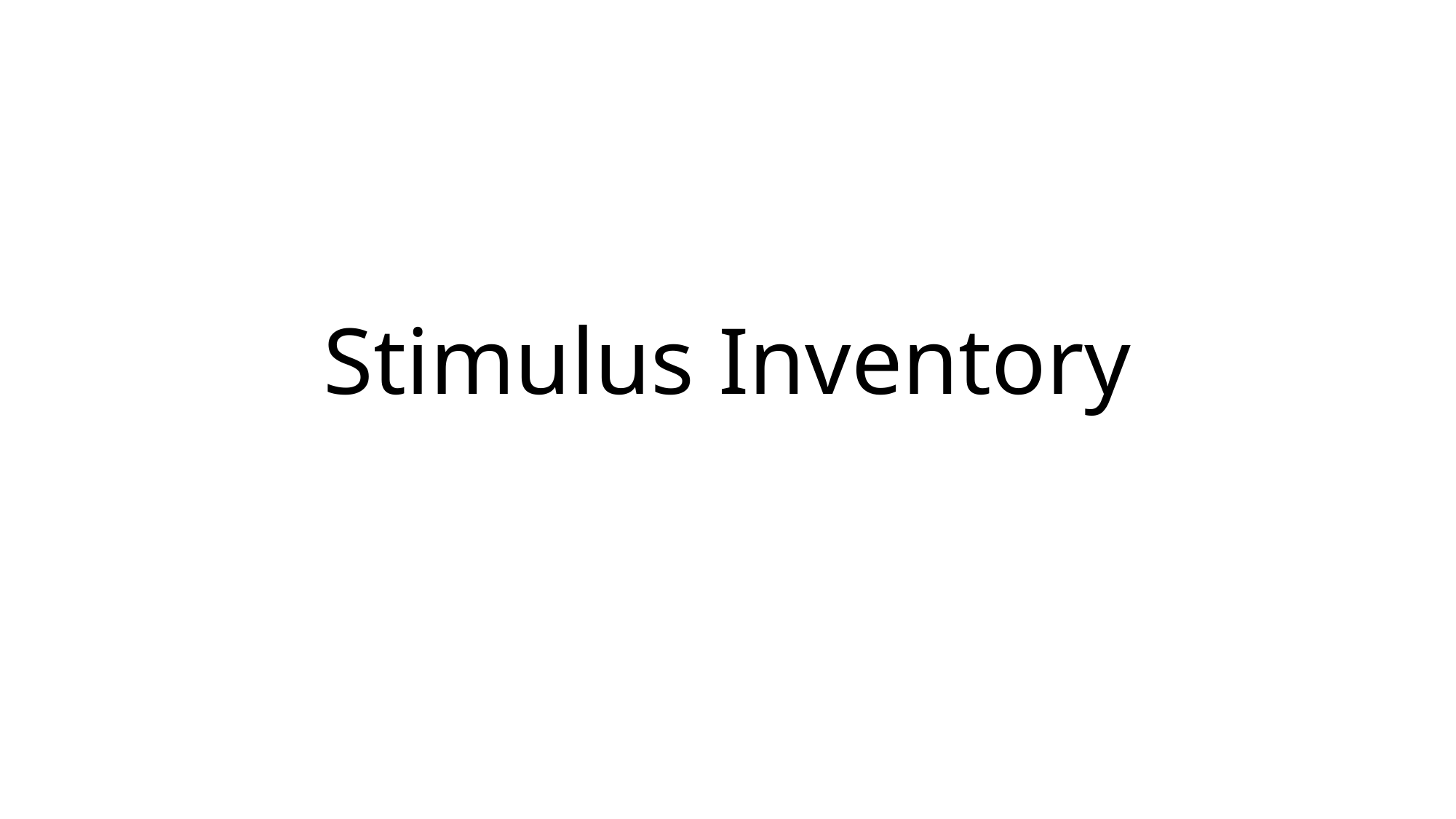

# Stimulus Inventory

## Slide 2
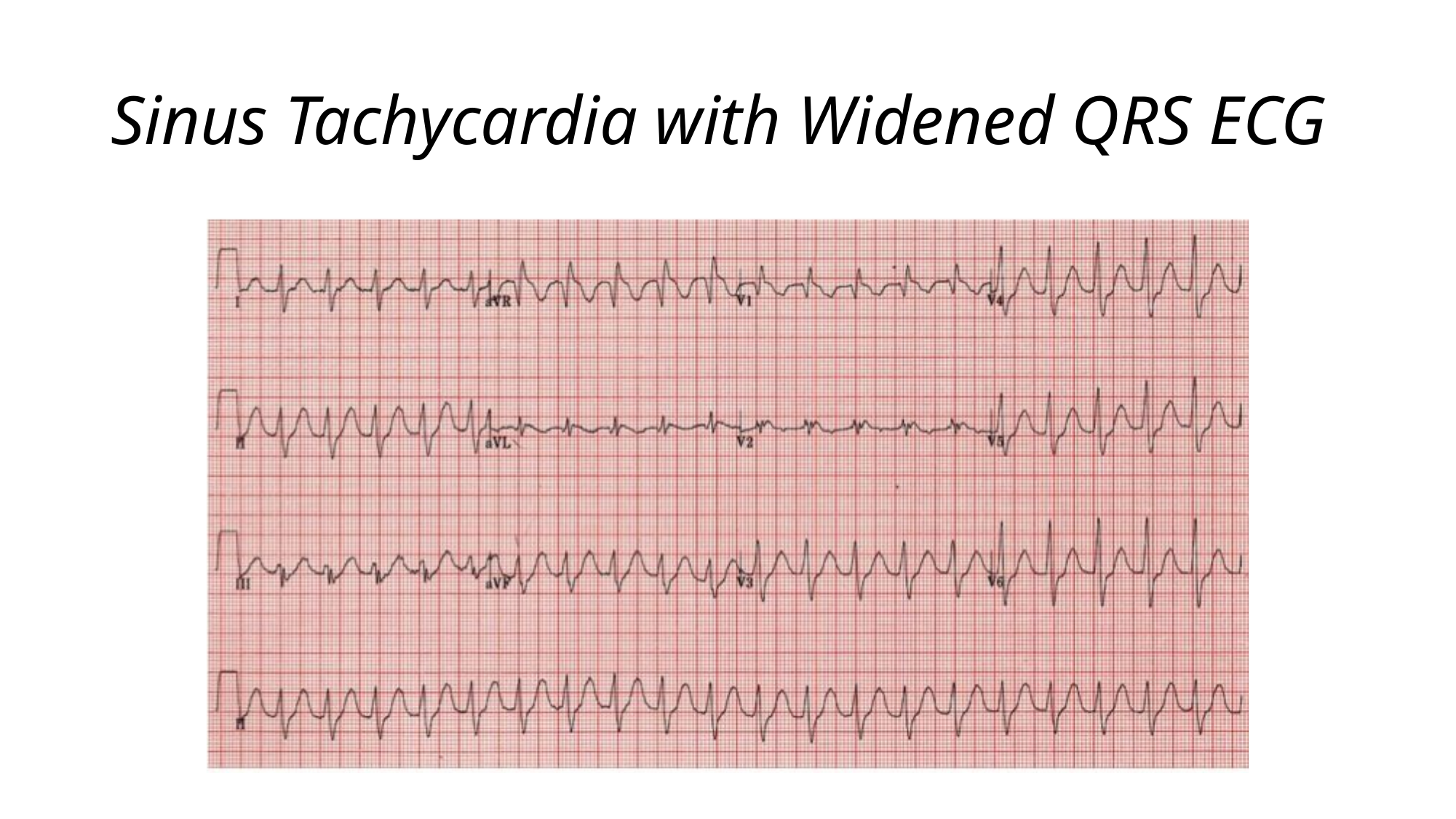

# Sinus Tachycardia with Widened QRS ECG

## Slide 3
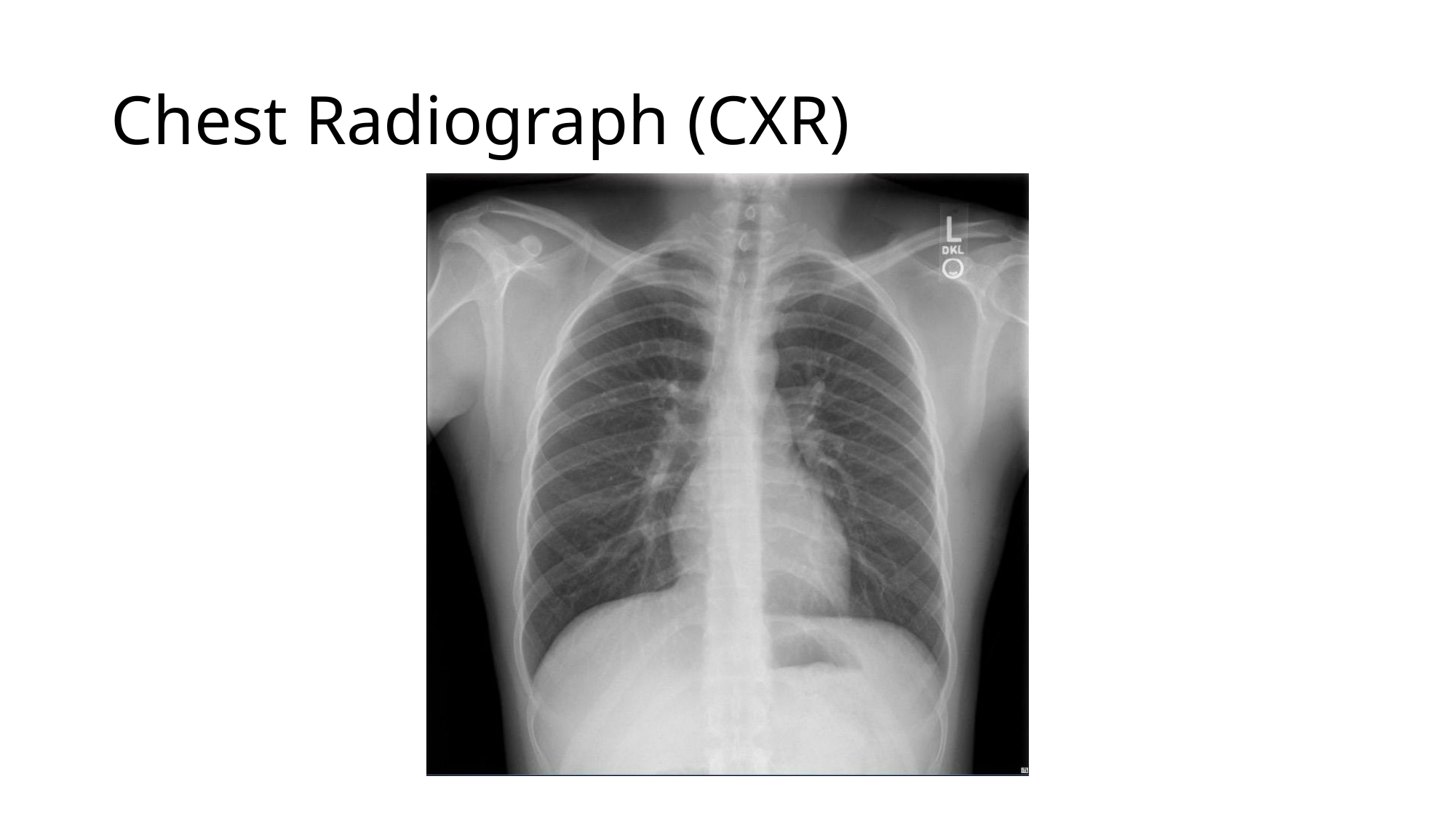

# Chest Radiograph (CXR)

## Slide 4
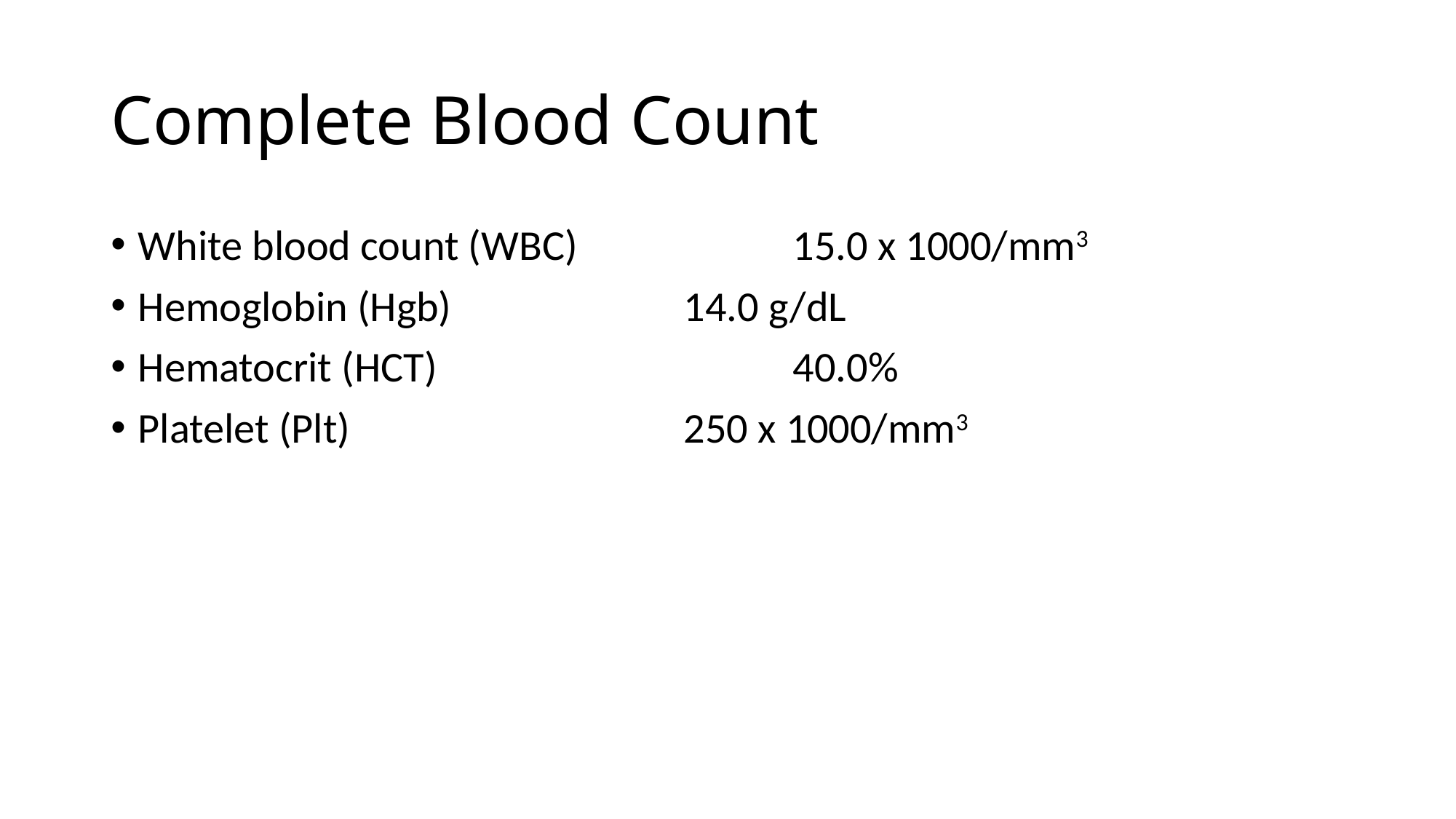

# Complete Blood Count
White blood count (WBC)		15.0 x 1000/mm3
Hemoglobin (Hgb)			14.0 g/dL
Hematocrit (HCT)				40.0%
Platelet (Plt)				250 x 1000/mm3

## Slide 5
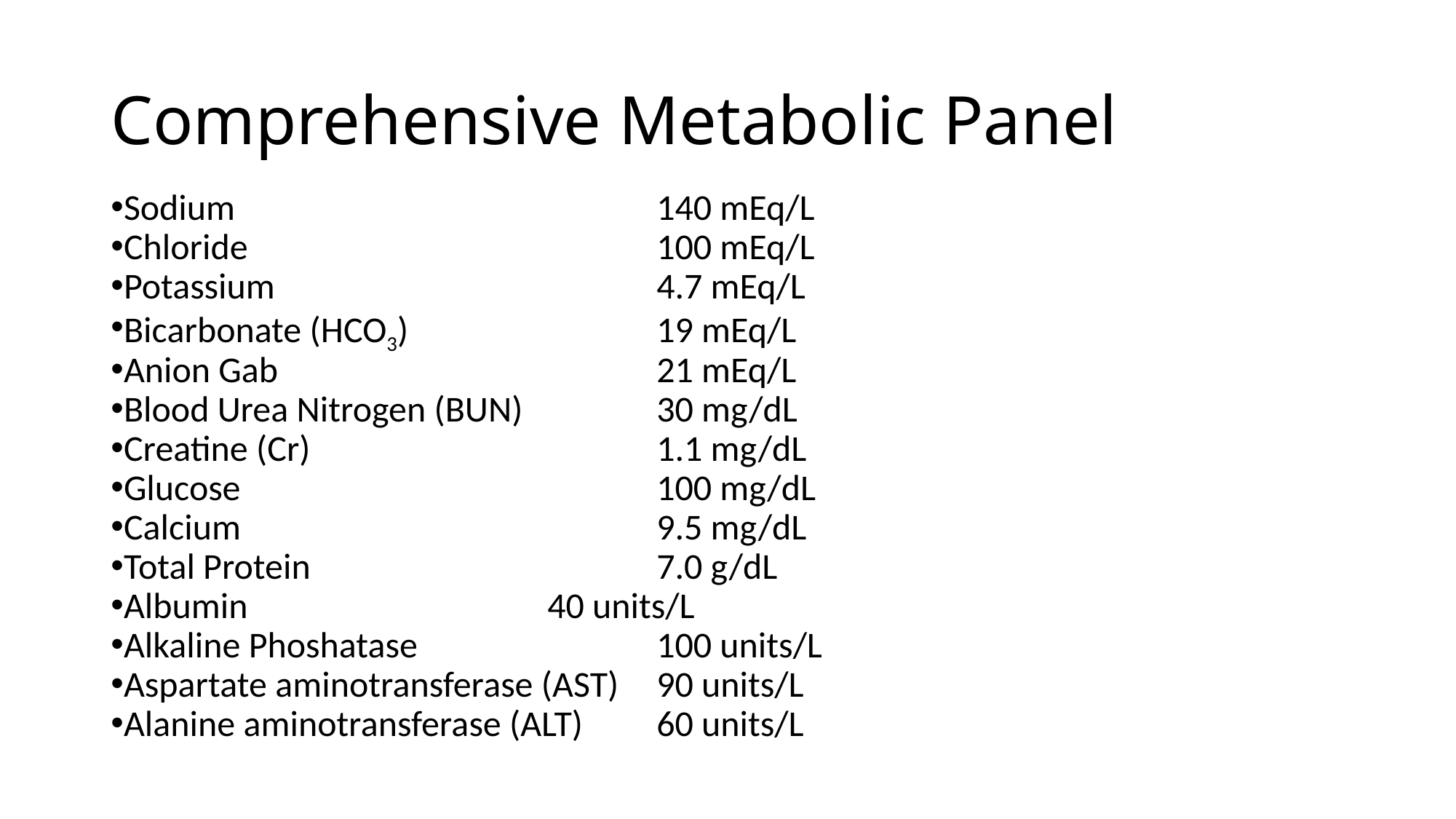

# Comprehensive Metabolic Panel
Sodium 				140 mEq/L
Chloride 				100 mEq/L
Potassium				4.7 mEq/L
Bicarbonate (HCO3)			19 mEq/L
Anion Gab				21 mEq/L
Blood Urea Nitrogen (BUN)		30 mg/dL
Creatine (Cr)		 		1.1 mg/dL
Glucose 				100 mg/dL
Calcium					9.5 mg/dL
Total Protein				7.0 g/dL
Albumin				40 units/L
Alkaline Phoshatase			100 units/L
Aspartate aminotransferase (AST)	90 units/L
Alanine aminotransferase (ALT)	60 units/L

## Slide 6
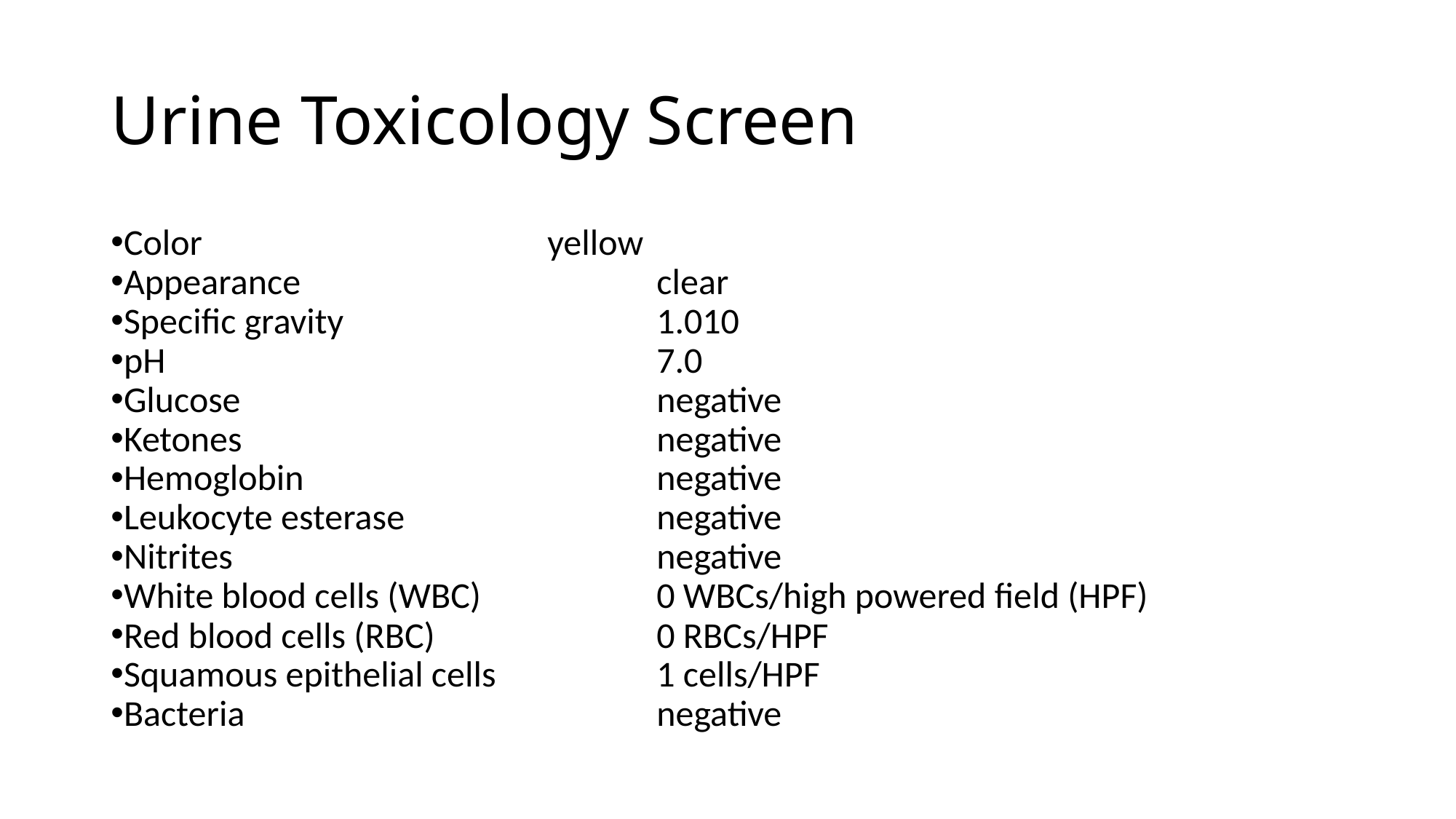

# Urine Toxicology Screen
Color 				yellow
Appearance 				clear
Specific gravity 			1.010
pH 					7.0
Glucose 				negative
Ketones 				negative
Hemoglobin				negative
Leukocyte esterase 			negative
Nitrites 				negative
White blood cells (WBC) 		0 WBCs/high powered field (HPF)
Red blood cells (RBC) 		0 RBCs/HPF
Squamous epithelial cells 		1 cells/HPF
Bacteria				negative

## Slide 7
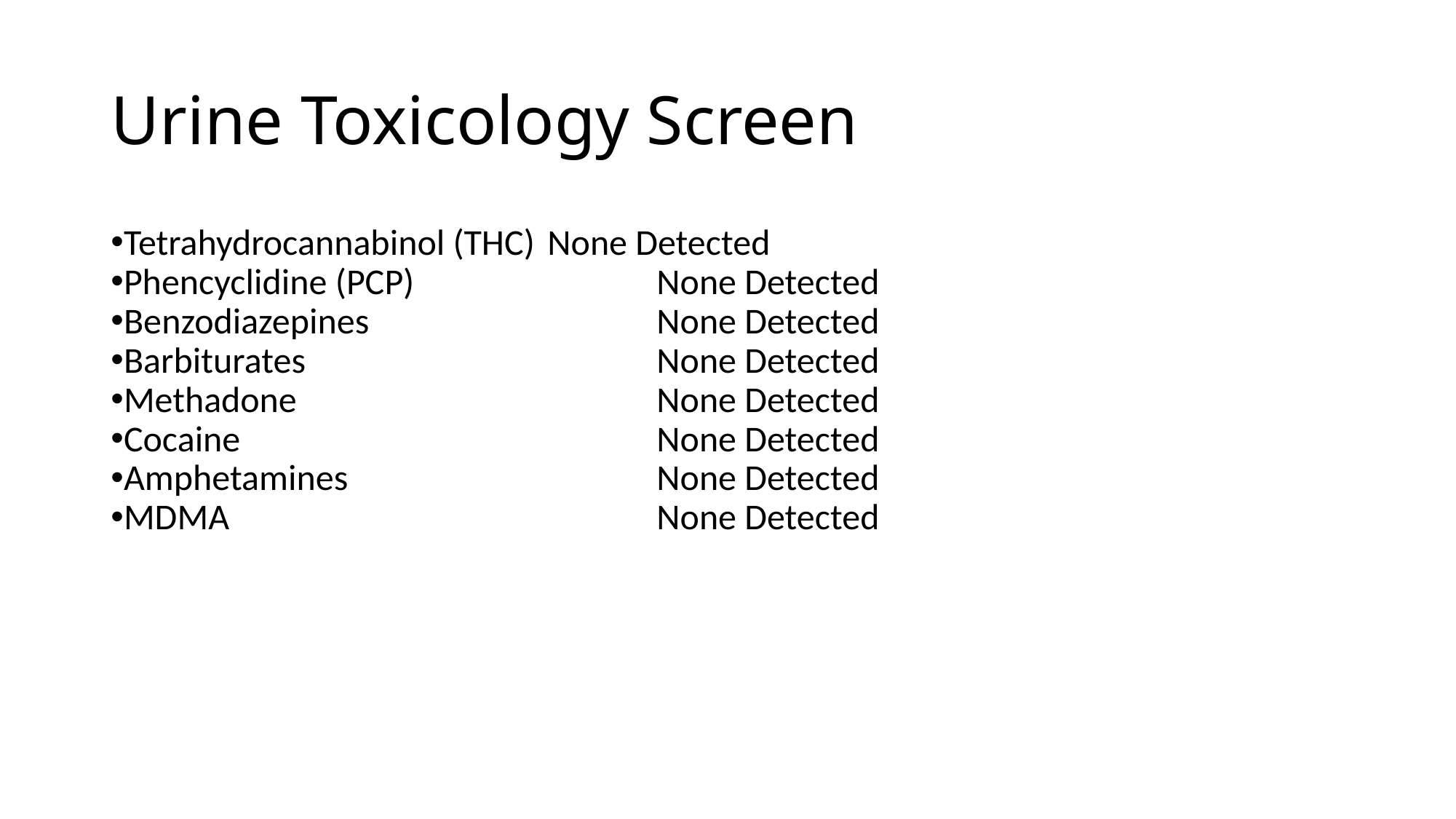

# Urine Toxicology Screen
Tetrahydrocannabinol (THC)	None Detected
Phencyclidine (PCP)			None Detected
Benzodiazepines			None Detected
Barbiturates				None Detected
Methadone				None Detected
Cocaine				None Detected
Amphetamines			None Detected
MDMA				None Detected
