# Supplementary figures and images for: Anticholinergic Toxicity in the Emergency Department
Source: J Educ Teach Emerg Med. 2023 Jan 31;8(1):S25–47. doi: 10.21980/J8D07Z (PMC10332772; doi:10.21980/J8D07Z)

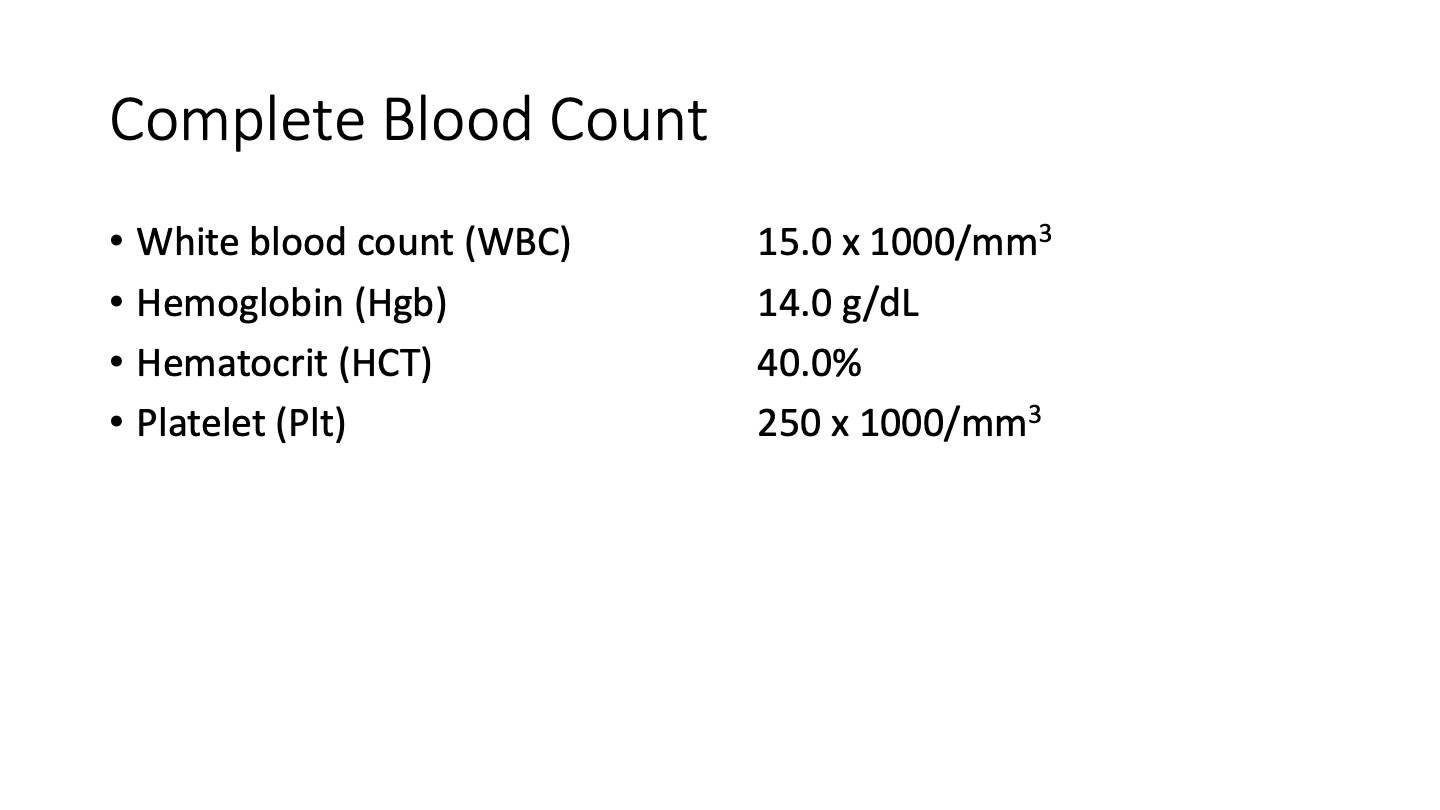

Supplement: Supplementary file 2 [file jetem-8-1-S25-supp2.jpg]

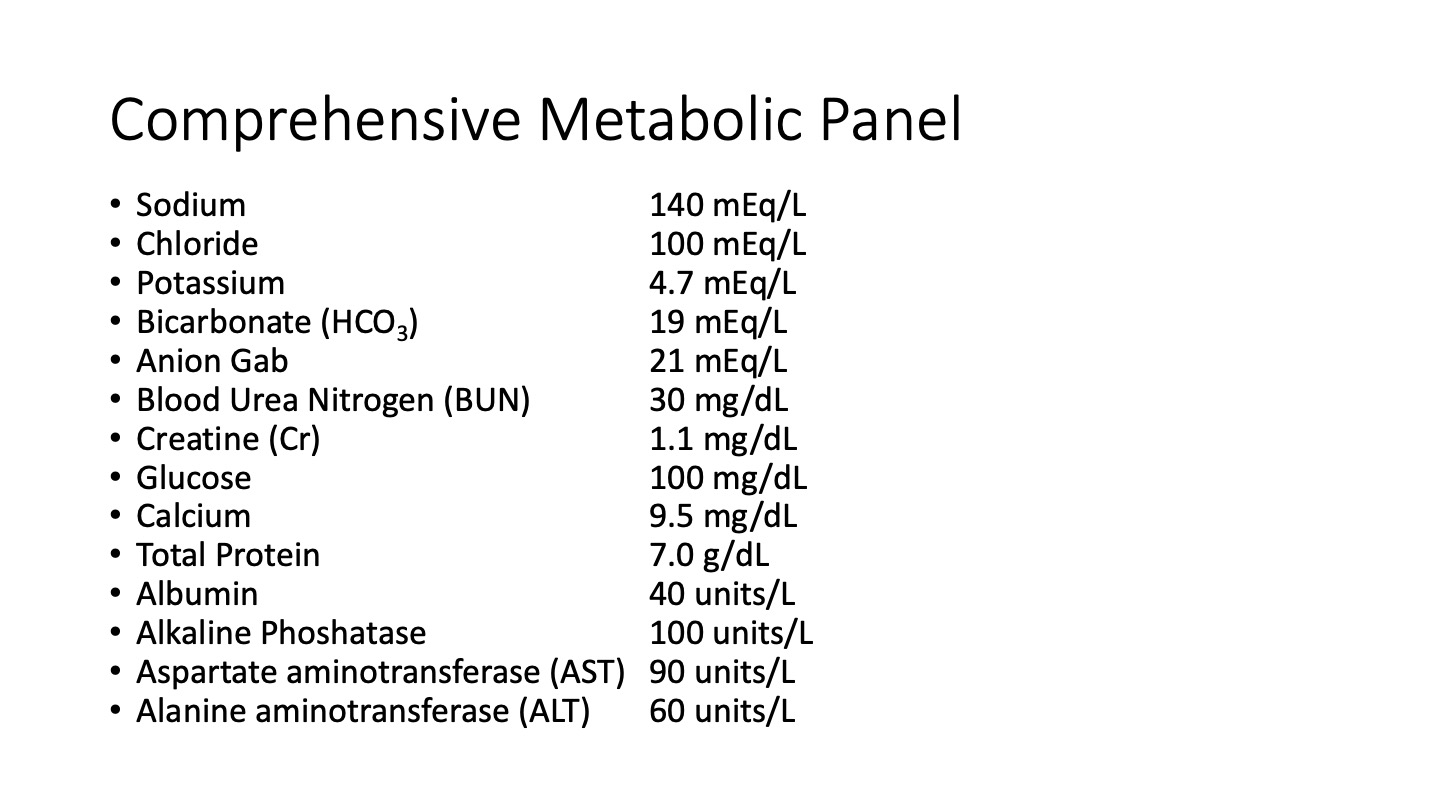

Supplement: Supplementary file 3 [file jetem-8-1-S25-supp3.jpg]

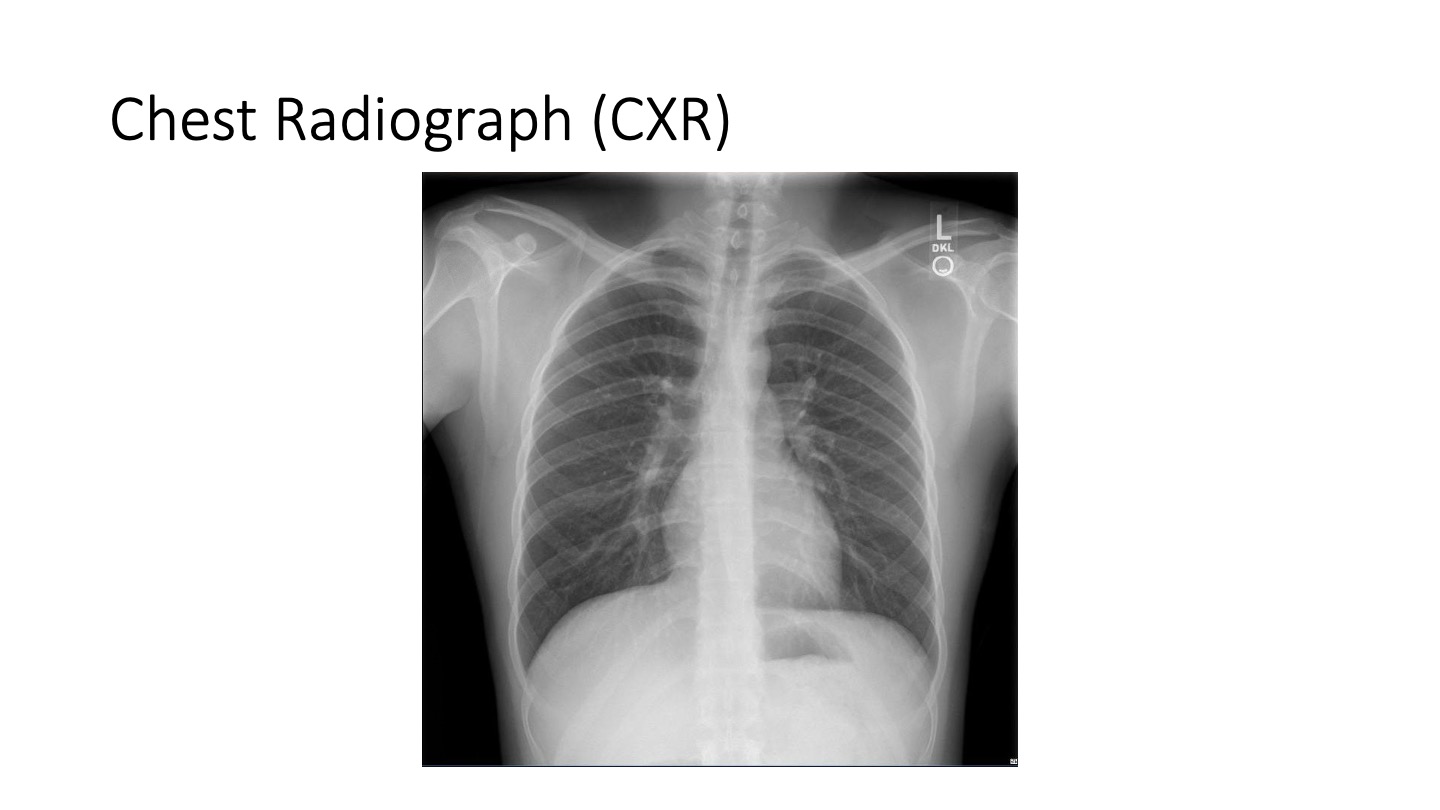

Supplement: Supplementary file 4 [file jetem-8-1-S25-supp4.jpg]

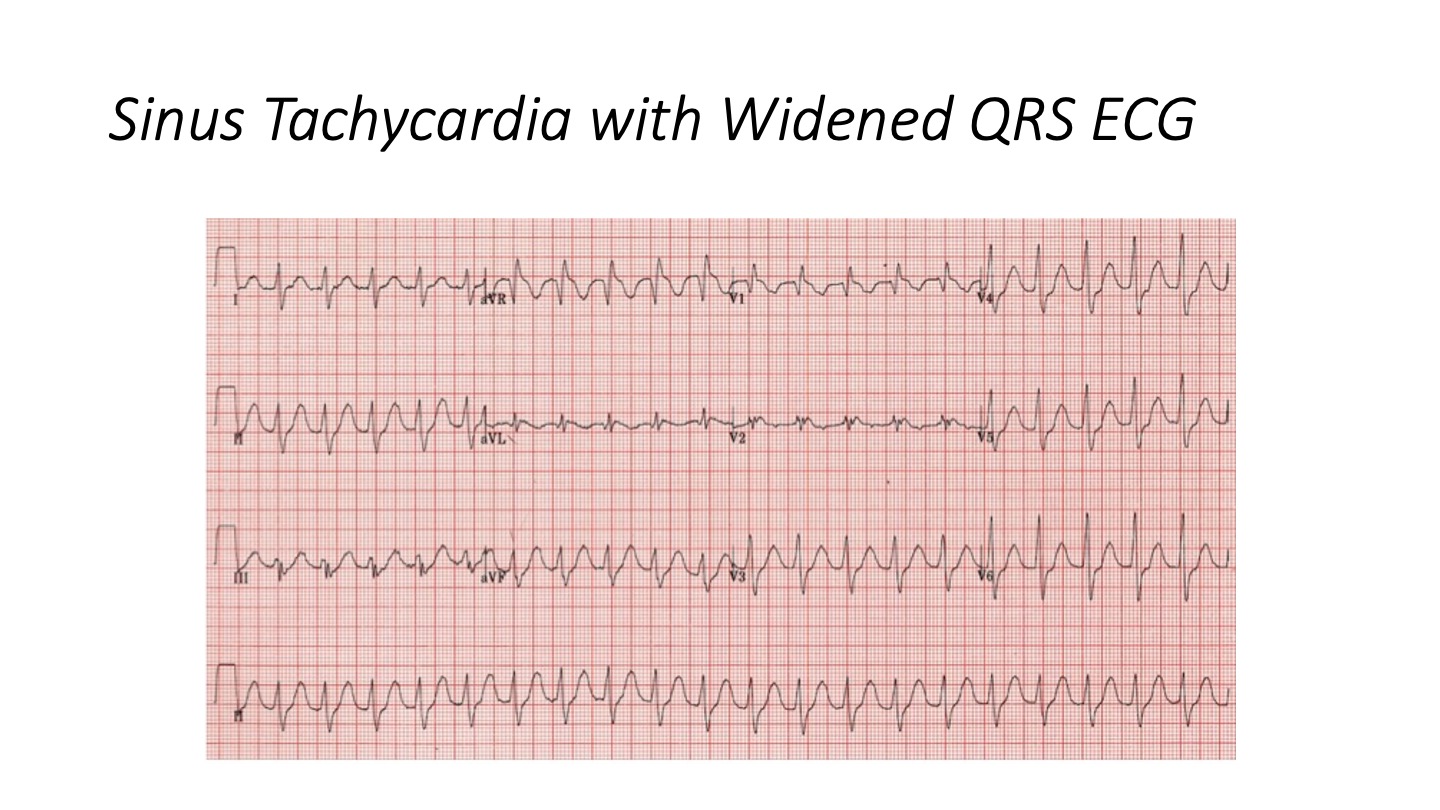

Supplement: Supplementary file 5 [file jetem-8-1-S25-supp5.jpg]

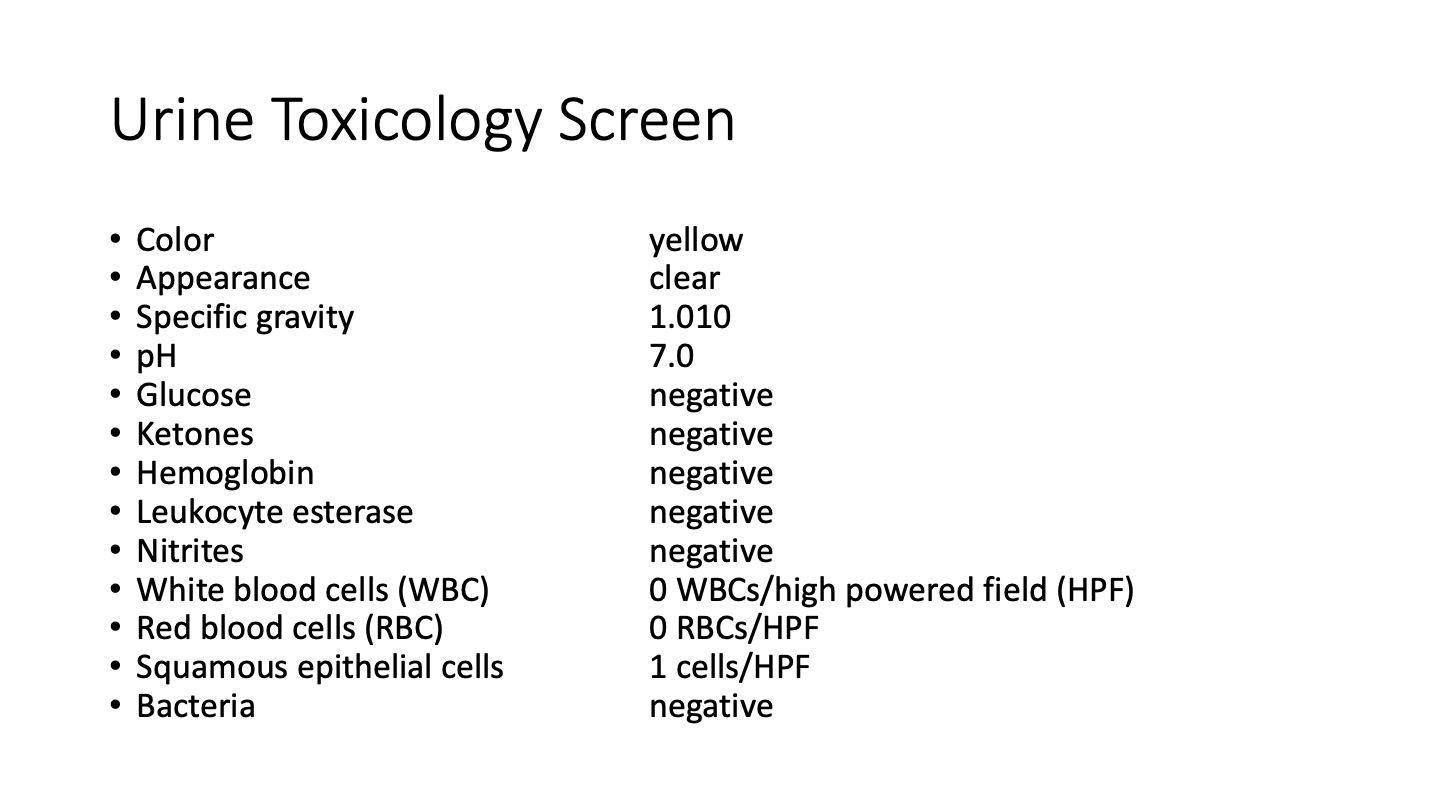

Supplement: Supplementary file 6 [file jetem-8-1-S25-supp6.jpg]

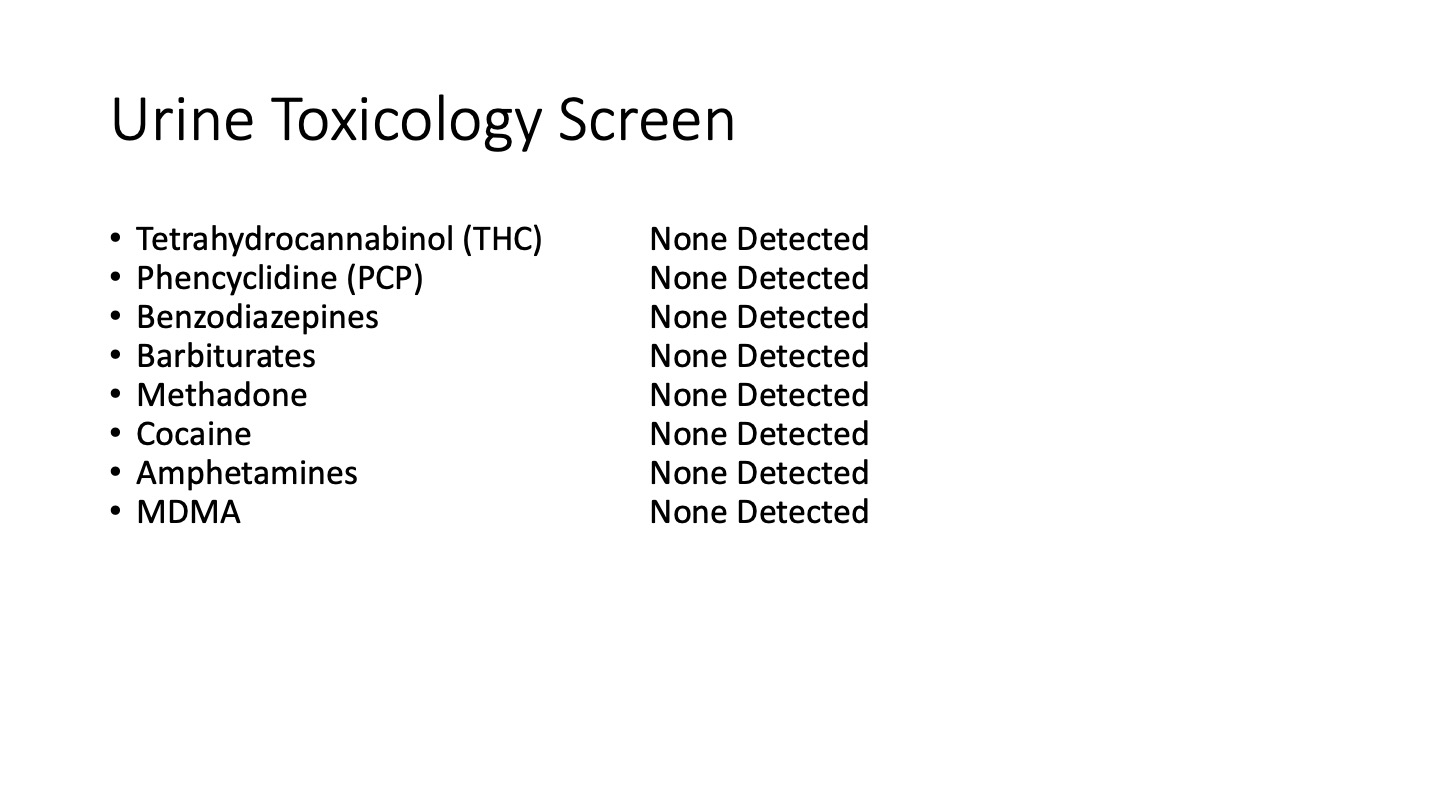

Supplement: Supplementary file 7 [file jetem-8-1-S25-supp7.jpg]
